# Hypovitaminosis-D: Frequency and association of clinical disease with biochemical levels in adult patients of RMI Medical OPD

**DOI:** 10.12669/pjms.322.9172

**Published:** 2016

**Authors:** Humaira Achakzai, Hammad Shah, Shahzada Bakhtyar Zahid, Muhammad Zuhaid

**Affiliations:** 1 Humaira Achakzai, MBBS, FCPS, Assistant Professor, Department of Medicine, Rehman Medical Institute, Peshawar, Pakistan; 2 Hammad Shah, MBBS, Resident Trainee FCPS, Department of Medicine, Rehman Medical Institute, Peshawar, Pakistan; 3 Shahzada Bakhtyar Zahid, MBBS, MRCPCH (UK), Associate Professor, Department of Pediatrics, Rehman Medical Institute, Peshawar, Pakistan; 4 Muhammad Zuhaid, MBBS, Resident Trainee, Department of Medicine, Rehman Medical Institute, Peshawar, Pakistan

**Keywords:** Hypovitaminosis D, Adults, Biochemical levels, Clinical severity

## Abstract

**Background and Objective:**

Vitamin D is steroid hormone essential for maintaining many important functions in the body. Hypovitaminosis D has become worldwide problem affecting all age groups and both genders, but its prevalence is very high in South Asia. Therefore this study was aimed to determine frequency of hypovitaminosis D in adult patients of RMI Medical Out Patients Department (OPD) and find association of clinical disease with biochemical levels.

**Methods:**

It was a cross sectional study carried out on all patients visiting RMI Medical OPD from 1^st^ Jan, 2015 to 30^th^ April, 2015 with clinical indications using universal sampling technique involving 400 subjects. Data was analyzed using SPSS 16.

**Results:**

Out of 400 subjects 85(21.3%) were males, 315(78.8%) were females, biochemical hydroxyvitamin-D3 deficiency was present in 320 (80%) subjects with 86(21.5%) having severe deficiency, 176(44%) having moderate deficiency and 58(14.5%) having mild deficiency. There was statistically significant association between biochemical deficiency and severity of clinical disease manifestation, calculated using chi square test. (p < 0.001).

**Conclusion:**

Most of the patient with musculoskeletal symptoms had vitamin D deficiency affecting all age groups and mostly female gender. There is a statistically significant association between low biochemical hydroxyvitamin-D3 levels and severity of clinical signs and symptoms which can provide evidence for replacement therapies in rural and far flung areas where biochemical assays are not available. Early recognition and replacement can prevent the massive complications, which deficiency of vitamin D makes us prone to develop.

## INTRODUCTION

Vitamin D is steroid hormone which is available in limited quantity in fortified dairy products and is synthesized mainly in the skin, from cholesterol, by exposure to the ultraviolet radiation of sunlight.^1^-^3^ Hydroxylation of vitamin D is done in the liver and kidneys, which is then transported to target tissues containing vitamin D receptors. Calcium homeostasis from the kidneys, bones, parathyroid gland, and intestine is maintained by vitamin D, playing a pivotal role in mineralization of bones.^4^ Vitamin D improves immunity, is essential for differentiation of cells and has established role in inhibition of cancer progression.^5^-^7^ It has a positive beneficial effect on cardiovascular and respiratory health.^8^-^10^ Brain health requires a normal Vitamin D level and its deficiency causes decrease in cognitive function of the brain.^11^,^12^ Normal vitamin D levels helps prevent pre-eclampsia during pregnancy and has significant anti-aging effect.^13^-^15^ Its deficiency causes myopathy, proximal muscle weakness with increased risk and tendency of falls and fractures.^16^-^19^

Hypovitaminosis D has become public health concern worldwide affecting all age groups.^20^,^21^ Its prevalence is very high in South Asia.^22^ South Asian population were deficient with mean level in severe Hypovitaminosis range when were their levels were checked in Denmark immigrants.^23^ There is generally lack of awareness among general population regarding beneficial effects of vitamin D on the body and with increasing urbanization and harshness of weather there is growing decline in people’s exposure to sunlight increasing deficiency of vitamin D in our population. Sedentary lifestyle and obesity contributes to low vitamin D3 levels because of decreased bioavailability from food and sunlight. Moreover cultural restraints, purdah observing in our society and increasing trend of using sun blocking creams further make the women of our locality vulnerable to hypovitaminosis D.

A study done on healthy adults in Karachi identified high prevalence of hypovitaminosis D in Pakistani healthy adults.^24^ But it focused mainly on healthy subjects and provided no details about its clinical signs, symptoms and severity of disease associated with vitamin D levels. No local standardized values are available for our population and a patient having hypovitaminosis with clinical manifestation in western population might be normal for our population. Another study was conducted in Lahore to identify prevalence of hypovitaminosis D in patients with musculoskeletal symptoms and a strong associated was found.^25^ It was not conducted in field to provide true prevalence of our community and all the patients with musculoskeletal pains with or without associated diseases were included which can be misleading to specify the true association of hypovitaminosis D with severity of clinical signs in our community.

This study was therefore designed to determine the frequency of hypovitaminosis D in adult patients visiting RMI medical OPD with clinical indication and to find association of biochemical levels with severity of signs and symptoms.

## METHODS

This study was carried out at Rehman Medical Institute(RMI), which is a tertiary care hospital in provincial capital Peshawar, providing specialized healthcare facilities to patients of different ethnic background, geographical location, socioeconomic status, all age groups and both genders. It was a cross sectional study, conducted on every patient visiting medical OPD clinic of RMI with clinical indication from 1^st^ Jan, 2015 to 30^th^ Apr, 2015, a period of four months. Universal sampling technique was used and patients from both sexes were included having age of more than 12 years. Those patients who had recently taken vitamin D supplements, already suffering from chronic liver disease, chronic kidney disease and had developed rickets, osteoporosis, osteoarthritis and osteopenia were excluded from the study. Out of 1368 patients presenting to medical OPD, 486 subjects had clinical indication, among whom 400 met the inclusion criteria and serum hydroxyvitamin D3 levels were determined with ELISA method using Architect SR 2000 machine and standard kit. Data was collected using a printed Performa. Informed consent was taken and confidentiality of information was ensured. This study was approved by research and ethics committee, after approval of research proposal by medical research committee of Rehman Medical Institute.

### Operational definitions

Clinical indication was defined as all those patients who had non-specific musculoskeletal aches and pains, arthralgia and bone pains in whom detailed history, clinical examination, laboratory serological and radiological investigations ruled out secondary etiology. Those patients who had recently taken multivitamin supplements were excluded by taking history using printed questionnaire. Patients with chronic liver disease were excluded by evaluating clinical signs of chronic liver disease involving clubbing, palmar erythrema, duputryen contracture, hair loss, gynaecomastia, spider naevi, cambel de morgan spots, enlarged liver span and spleen, presence of ascites and confirmation by ultrasonographic evidence of heterogenous parenchymal echotexture of liver regardless of the etiology contributing or isolated ultrasonographic evidence without clinical signs. Patients with chronic kidney disease were excluded by evaluation of symptoms of nausea, vomiting, dyspnea having signs of fluid overload like pulmonary congestion clinically and radiologically on chest X-Ray with a normal systolic function on echocardiography, deranged biochemical renal profile with raised serum creatinine and urea than normal and presence of ultrasonographic evidence of renal parenchymal disease or isolated ultrasonographic evidence of renal parenchymal disease with deranged biochemical profile or patients on renal supportive therapy of dialysis. A serum creatining of >2mg/dl and serum urea of >60mg/dl with Glomerular Filtration Rate of < 40 was termed as abnormal when repeated 48 hours apart not responded to conservative treatment of isotonic fluid infusions and remained high. Bone diseases were excluded by taking history of established bone disease or having generalized osteopenia in X-ray of long bones with reduced joint spaces, erosions or bone deformities.

Mild symptoms were defined as non-specific aches and pains without involvement of bones, moderate symptoms were defined as non-specific aches and pains with bone involvement without affecting routine daily activities. Severe symptoms were defined as non-specific aches and pains with bone involvement which affected daily activities.

Severe hypovitaminosis was defined as serum 0H Vit-D3 level of 0-9.9ng/ml, moderate hypovitaminosis was defined as serum OH Vit-D3 levels of 10-19.9ng/ml and mild hypovitaminosis was defined as serum 0H Vit-D3 level of 20-29.9ng/ml. Normal vitamin D levels were defined as having serum OH vit d3 levels of 30-100ng/ml.

## RESULTS

A total of 400 patients with 85 (21.3%) males and 315(78.8%) females were studied. Continuous variables like age and serum hydroxyvitamin D3 levels were determined as (±standard deviation) having mean age of 41.34(±14.77) and mean hydroxyvitamin D3 level of 24.53 (±28.79) as shown in Table-I.

**Table-I:** Descriptive analysis of age and Hydroxyvitamin D3 levels.

	Number	Mininum	Maximum	Mean	Standard Deviation
Age	400	13	96	41.34	14.77
Hydroxy Vitamin D3 Level	400	1	160	24.53	28.79

Biochemically hypovitaminosis D was present in 80% (320 patients) of population with 19% (76 patients) males and 61% (244 patients) females having a male to female ratio of 1:3.2. Severe hydroxyvitamin-D3 deficiency was present in 21.5% (86 patients) out of which 5.25% (21 patients) were males and 16.25% (65 patients) were females, Moderate hydroxyvitamin-D3 deficiency was present in 44% (176 patients) with 11.5% (46 patients) males and 32.5% (130 patients) females. Mild hydroxyvitamin-D3 deficiency was present in 14.5% (58 patients) with 2.25% (09 patients) males and 12.25(49 patients) females. Biochemical deficiency was not present in 20% of population with clinical indication out of which 16.3% (65 patients) had normal vitamin D levels with 2.25% (09 patients) males and 14.05% (56 patients) females. A total of 3.8% (15 patients) patients had more than normal values all of whom were females as shown in Table-II.

**Table-II:**
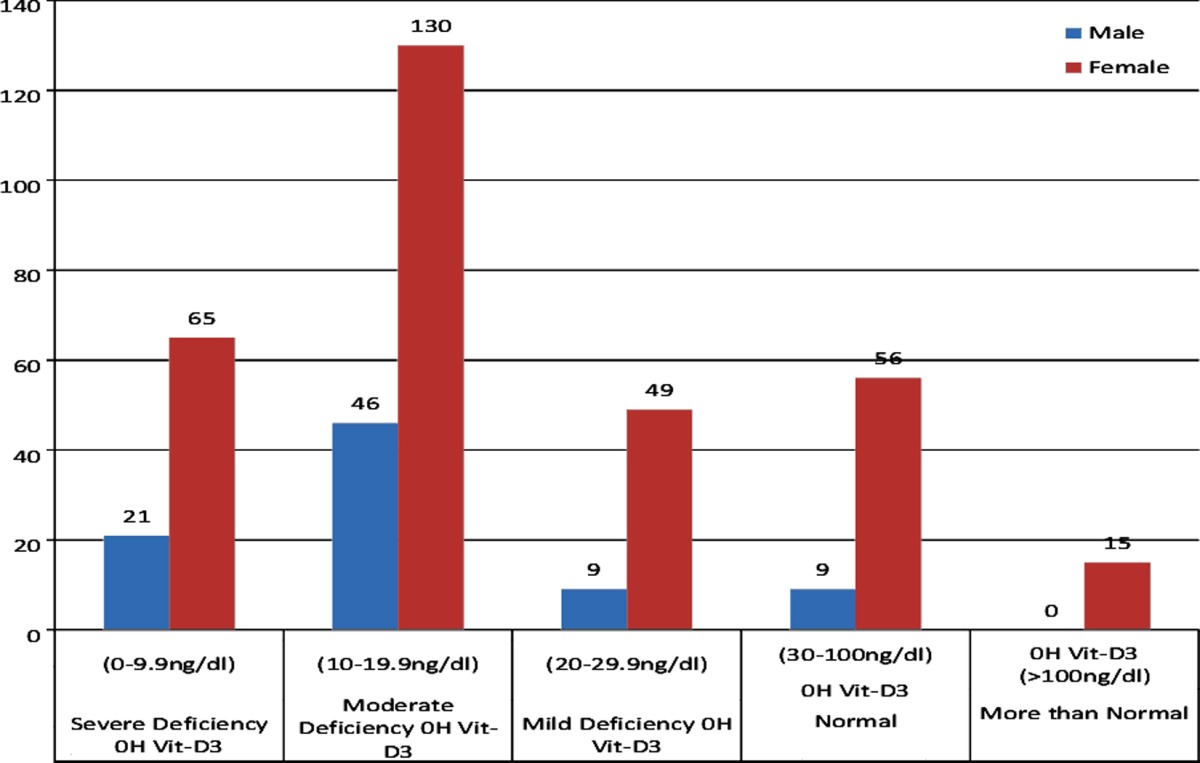
Hydroxy-vitamin D3 interpretation in relation to gender differences.

**Table-III:** Hydroxy-Vitamin D3 levels association with clinical severity of symptoms.

Biochemical Levels		Clinical Severity of symptoms				P value

	Mild Symptoms	Moderate Symptoms	Severe Symptoms	Total	
Severe Deficiency 0H Vit-D3(0-9.9ng/dl)	1	16	69	86
Moderate Deficiency 0H Vit-D3 (10-19.9ng/dl)	12	156	8	176
Mild Deficiency OH-Vit D3 0H Vit-D3 (20-29.9ng/dl)	56	2	0	58
Normal 0H Vit-D3(30-100ng/dl)	64	1	0	65
More than Normal 0H Vit-D3 (>100ng/dl)	15	0	0	15

Total	148	175	77	400

Severe hydroxyvitamin-D3 deficiency was present in 21.5% (86 patients) out of which one patient had mild symptoms,^16^ had moderate symptoms and 68 had severe symptoms, Moderate hydroxyvitamin-D3 deficiency was present in 44% (176 patients) out of which 12 patients had mild, 156 had moderate and 12 patients had severe clinical symptoms. Mild hydroxyvitamin-D3 deficiency was present in 14.5% (58 patients) out of which 56 patients had mild clinical symptoms and two patients had moderate symptoms with no patient having severe symptoms. Biochemical deficiency was not present in 20% of population with clinical indication out of which 16.3% (65 patients) had normal vitamin D levels with 16.04% (64 patients) having mild symptoms and only 0.25% (one patient) having moderate symptoms and no patient having severe symptoms. A total of 3.8% (15 patients) patients had more than normal value with no patient having moderate or severe symptoms. There was statistically significant association between serum hydroxyvitamin D levels with clinical signs and symptoms using chi square test. (p< 0.001).

## DISCUSSION

Vitamin D deficiency has become global public health concern and our study showed that it affected patients of all age groups. this finding is congruent to other studies conducted in two different regions.^21^,^22^ Despite having opportunity of adequate sunlight exposure all year around our study showed a large proportion of our patients (80%)had low levels of vitamin D and there was a strong positive association with female gender. Similar findings are shown by various other studies conducted on South Asian population.^21^,^22^,^23^ This is explainable because of traditional norms and cultural restraints of our population. Female observe purdah, are confined to their houses mostly, lives sedentary lifestyles and are dependent on males for outside needs fulfillment. Moreover there is growing trend in females of our society, using sun-blocking creams, which further make them vulnerable to be affected. There is strong cultural restraint of darkening of skin with sun exposure and people intentionally avoid exposure to sunlight. All these factors are contributing to disease burden in our society.

Low vitamin D levels manifests itself as musculoskeletal signs and symptoms as shown by results of various national and international studies.^25^,^26^,^27^ Our study targeted those patients and provided association between severity of signs and symptoms with biochemical values and it was evident that in 80 % of patients, severity of symptoms was strongly associated with biochemical levels. However 20% patients had clinical indication with no vitamin D deficiency and most (98.8%) of these patients had only mild symptoms of non-specific musculoskeletal aches and pains without involving bones which could be due to other causes like fibromyalgia’s, refractory musculoskeletal pain and unusual pain syndromes.^27^ Strength of the study was that 99.4% of the patients who had moderate symptoms and all the patients with severe symptoms had very low serum hydroxyvitamin D3 levels leading to recommendation that we can apply corrective replacement therapies in rural and far flung areas where biochemical assays are not available. Early recognition and prompt replacement will help us to avoid multisystemic side effects which we are prone to develop due to its deficiency.

### Limitations of the study

It was a cross sectional hospital based study, the results cannot be truly projected to general population but it will pave wave for similar population based epidemiological studies in our locality. Since the patients were included using clinical signs and symptoms we might have missed the asymptomatic individuals who were actually diseased.
